# Epidemiological Risk Factors and Modelling Approaches for Risk Assessment of Lumpy Skin Disease Virus Introduction and Spread: Methodological Review and Implications for Risk-Based Surveillance in Australia

**DOI:** 10.1155/2024/3090226

**Published:** 2024-05-02

**Authors:** Kei Owada, Timothy J. Mahony, Rebecca K. Ambrose, Ben J. Hayes, Ricardo J. Soares Magalhães

**Affiliations:** ^1^Queensland Alliance for One Health Sciences, School of Veterinary Science, The University of Queensland, Gatton 4343, Australia; ^2^Centre for Animal Science, Queensland Alliance for Agriculture and Food Innovation, The University of Queensland, St Lucia 4072, Australia; ^3^Department of Agriculture and Fisheries, Queensland Government, Brisbane 4000, Australia; ^4^Children's Health and Environment Program, Children's Health Research Centre, The University of Queensland, South Brisbane 4101, Australia

## Abstract

Lumpy skin disease (LSD) is a vector-borne infection caused by the poxvirus lumpy skin disease virus (LSDV) and is a serious disease of cattle, water buffalo, and banteng. While the disease has never occurred in Australia, it is regarded as a growing threat to the Australian cattle industry as there is on-going spread of the disease throughout Asia. The development of geospatial decision support tools, such as spatial epidemiological modelling, may assist in assessing areas at greater risk of this threat. To guide the design of disease modelling approaches to support future risk-based surveillance, existing LSDV epidemiological models need to be evaluated. In this study, we performed a literature review to evaluate existing LSDV epidemiological models, identify key risk factors for introduction and spread of LSDV, and consider previously adopted control strategies. The PRISMA guidelines were used to establish the processes for article selection and information extraction, and the PICO process was used to formulate search terms. From studies that met our inclusion criteria, we extracted information on LSDV epidemiological model structure and parameterisation, risk factors for LSDV transmission and spread, and biosecurity control strategies. The literature search retrieved a total of 402 articles from four databases, of which 68 were identified for inclusion in this review following screening. Of the 68 articles reviewed, 47 explored risk factors associated with LSDV transmission and spread, four explored risk factors of LSDV introduction, four explored existing surveillance strategies in LSD-free countries, and 14 presented epidemiological models. Our findings indicate that there are various risk factors for LSDV transmission in LSD endemic countries, including long-distance airborne movement of infected vectors such as stable flies and cattle movement between countries over land borders. Key risk factors for LSDV spread in LSD endemic countries include physical environmental characteristics, weather conditions, and population distributions of livestock and vectors. Our results indicate that while a variety of modelling studies have been conducted, the majority of studies experimentally explored LSD transmission mechanisms in vectors and cattle. Spatial and spatio-temporal models have primarily been developed for LSD endemic countries and focus on the spread of the disease in terms of environmental factors in relation to previous LSD events. There were very few studies on LSD-free countries, and these only focussed on risk of LSD introduction through specific entry pathways. This review did not identify any literature exploring the risk of spread of LSDV following introduction in LSD-free countries or geospatial modelling of the suitability of LSD-free countries for LSDV incursions. In conjunction with the risk parameters and models described in the identified literature, there is need to consider a wide range of risk factors specific to Australia to inform the design of risk-based surveillance for LSD in Australia.

## 1. Introduction

Lumpy skin disease (LSD) is an enzootic arthropod-borne viral disease caused by the lumpy skin disease virus (LSDV) that primarily affects cattle (*Bos indicus* and *Bos taurus*) and water buffalo (*Bubalus bubalis*), with recent reports of it also affecting banteng (*Bos javanicus*) [1–3]. LSDV belongs to the genus *Capripoxvirus*, within the subfamily *Chordopoxvirinae* of the family *Poxviridae*. To date, LSDV infection has not been reported in sheep and goats, who are susceptible to sheep and goat pox viruses, which are also members of the *Capripoxvirus* genus [2]. The incubation period has been estimated to be between 1 and 4 weeks. Early signs of infection include fever in the range of 40–41°C and increased lachrymation. Following onset of fever, multiple skin nodules, approximately 1–5 cm in diameter, appear, which may cover the animal's entire body. The morbidity rate can vary widely from 3% to 85% and is dependent on host immunity and prevalence of arthropod vectors [1, 2]. The mortality rate is usually below 10% [4]. LSD results in reduced milk production and can also lead to temporary or permanent infertility in both cows and bulls [1]. LSD is a World Organization for Animal Health (WOAH)-listed disease [1] due to its significant economic impacts through livestock production losses and international trade restrictions [1, 5–8]. To address the impact of LSD, organisations such as the DEFEND consortium are actively researching LSD in order to develop new prevention and control measures [9]. Biting insects, including flies, mosquitoes, midges, and ticks, are considered the most likely vectors of LSDV, with experimental evidence suggesting that *Aedes aegypti* mosquitoes and stable flies (*Stomoxys calcitrans*) are capable of transmitting LSDV [2, 10, 11]. The most common approaches to controlling the spread of LSD include quarantine and movement restrictions, removal/culling of infected animals, tracing and surveillance, vector management, and vaccination [2].

LSD was first reported in Zambia in 1929 and is now considered endemic throughout most of Africa [7]. LSD was first reported outside of Africa in Israel in 1989 and has since spread into other countries in the Middle East [7]. The first LSD outbreaks in Europe were recorded in eastern Europe in 2015; however, the spread of the disease has been mostly controlled through the deployment of live-attenuated homologous vaccines [12, 13]. In 2019, LSDV spread into east and southern Asia, with outbreaks recorded in China, India, and Bangladesh [4], and has since spread into multiple South-east Asian countries [14]. In 2022, LSDV was reported in Indonesia and has spread across the Indonesian islands of Sumatra and Java, with the disease most recently reported in East Java in December 2022 [15]. As of 19 May 2023, LSD has been confirmed in 15 provinces of Indonesia, including Aceh, North Sumatra, West Sumatra, South Sumatra, Riau, Jambi, Bengkulu, Lampung, Banten, West Java, Central Java, East Java, Yogyakarta, and Central Kalimantan province [16]. As of April 2024, LSD has never occurred in Australia [17].

With the spread of LSDV coming into close geographical proximity to Australia, there is a need to assess the risk of LSDV reaching mainland Australia. An incursion of LSDV into Australia would likely result in the introduction of restrictions to exports of both live cattle and meat and dairy products [18]. This could have a significant economic impact on Australia's cattle industries, with beef exports in 2021 valued at A$9.2 billion [19] and dairy exports in 2021–2022 (36% of annual milk production in Australia) valued at A$3.8 billion [20]. In 2021, Australia exported 771,931 live cattle, of which 53% were sent to Indonesia [21].

A recent risk assessment based on qualitative and quantitative models determined the overall risk of LSDV incursion into Australia to be very low when assuming 3–5 vectors are required for successful vector-to-bovine LSDV transmission and negligible when assuming at least 30–50 vectors are required for successful LSDV transmission. However, the risk assessment also highlighted that gaps in the understanding of LSDV transmission hamper the accurate estimation of actual risk of LSDV incursion [22, 23]. Also, while the overall risk of incursion is expected to be negligible to very low, it is likely that there are areas in the country where the risk of incursion is highest, requiring risk-based approaches for their identification. In order to address these gaps, geospatial decision support tools, such as spatial epidemiological modelling, may be applied to investigate the effect of a wide range of LSDV risk factors, including climate and environmental factors and the existence of sub-national areas where the risk of incursion and spread is likely to be highest [24]. In the context of the current Australia risk profile for an LSDV incursion, biosecurity threat assessments supplemented by geospatial decision support tools are needed to inform evidence-based biosecurity prevention and response preparedness to a possible LSDV incursion in Australia.

This scoping literature review has two aims: (1) to identify available evidence on risk factors influencing the introduction and dissemination of LSDV in livestock populations, risk factors for LSDV exposure and infection of cattle, and LSD control strategies and (2) to review existing epidemiological modelling approaches implemented. The findings of this review will provide a basis for designing of spatial epidemiological disease modelling approaches to inform control and prevention of LSD in Australia.

## 2. Methods

### 2.1. PICO Process

The PICO literature review process [25] was applied to formulate search terms to be used on literature databases by extracting keywords based on the aims using four components: population or problem, intervention or exposure, comparison, and outcome. Details of the PICO process used are detailed in *Supplementary 1*.

### 2.2. Inclusion Criteria

This literature review did not limit search results to any specific geographical area or year range. The literature review included literature written in any language. The investigators for this literature review were able to read and validate contents of literature written in English, French, Spanish, Portuguese, and Japanese. Literature written in other languages were reviewed provided accurate machine translation was available. Grey literature, including unpublished and pre-print articles, were also included in this review. The following study types were included in the literature review: observational, cohort, spatial epidemiology, mapping, and mathematical modelling.

### 2.3. Exclusion Criteria

The following search terms were included in database searches with the NOT Boolean operator and searching only in titles and abstracts: clinical trial, randomised control trial, vaccination, vaccine, genomic, genome, phylogeny, and biochemical.

### 2.4. Search Strategy

This literature review searched for available literature using five databases: PubMed, Scopus, Web of Science, Europe PMC, and the University of Queensland library website. All five databases were searched, and their results were recorded on 6 March 2023. The keyword searches used are detailed in *Supplementary 1*.

### 2.5. Screening

**Figure 1:**
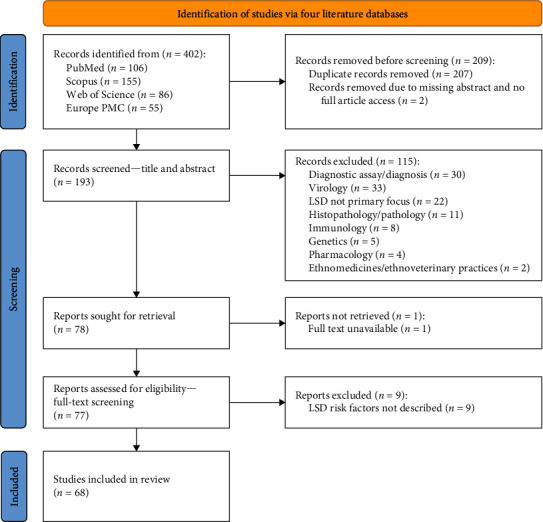
PRISMA flowchart of search and screening of literature.

Search results were first screened for duplicate results as detailed in the PRISMA flowchart (Figure 1) [26]. The remaining results were then screened based on titles and abstracts, to ensure that contents of the results relate to at least one of the aims of the literature review. The number of results that were discarded as a result of each screening process was recorded, along with the reasons for exclusion.

### 2.6. Data Extraction and Storage of Results

All search results were recorded in EndNote software, and data retrieval for full-text reviewed papers was conducted using a Microsoft Excel spreadsheet (*Supplementary 2*).

### 2.7. Quality Assessment

Articles that passed the screening stage were read in full to assess the relevance of their contents, as well as the validity of their study methods and results.

## 3. Results

A total of 402 records were retrieved from the four literature databases. After screening, a total of 68 studies met the selection criteria and were selected for full in-text review (Figure 1). The contents of the 68 studies identified have been critically evaluated under the five main themes described in Section 2.6 [1, 4–8, 10, 11, 13, 14, 27–84]. Citations in this review to literature other than these 68 studies correspond to additional resources that were drawn upon to provide supporting evidence to assist in the discussion of the risk of an LSDV incursion into Australia. The details of the reviewed articles can be found in the references section at the end of this document.

### 3.1. Global and Regional Epidemiology of LSD

LSD was first recorded in Zambia in 1929 [7]. Since then, LSD has spread through Africa, including Kenya [10], Nigeria [53], Ethiopia [1, 8, 27, 30, 38, 41, 43], Uganda [66, 68], South Africa [37], and Egypt [37, 51, 77]. LSDV infections have also been detected sporadically in parts of the Middle East (since 1989) and Europe and Asia (since 2015), including Albania [52, 61], Greece [13, 64], Bulgaria [13, 64], North Macedonia [13, 64], Kazakhstan [47], Russia [13, 14], Armenia [13], Türkiye (formerly Turkey) [5, 7, 13, 14, 29, 46, 61, 78], Israel [13, 51, 74, 84], Lebanon [7], Jordan [7, 13, 28], Iraq [7, 29], Saudi Arabia [13], and Oman [80]. Since 2019, the disease has emerged in South and South-east Asia, including large outbreaks reported in India [6, 33, 35, 60, 69], Bangladesh [6, 33, 35, 60], Pakistan [6, 45, 50], Sri Lanka [33, 35], China [4, 45, 57], Hong Kong [6], Taiwan [33, 57], Bhutan [6, 33, 35], Nepal [6, 33, 35, 60], Vietnam [6, 14, 33, 35, 60], Laos [14, 35], Cambodia [14, 35], Myanmar [6, 14, 33, 35, 60], and Thailand [6, 7, 14, 33, 35, 64]. Additionally, while no reports were contained in the results of the literature review, LSDV was first reported in Indonesia in March 2022 via the World Organisation for Animal Health, and most recently in East Java in December 2022 [18, 85]. A number of countries are still considered to be LSD-free, including Australia and the United Kingdom [86].

### 3.2. LSDV Transmission and Vectors

Mosquitoes were first implicated as a possible vector for LSDV following an LSDV outbreak in Kenya in 1959 due to the infestation of Culicidae species in the outbreak areas [10, 70]. The first experimental test to confirm biting insects as a LSDV vector found *A. aegypti* to be an efficient vector, with the species being able to transmit LSDV to cattle up to 6 days following feeding on LSDV-infected cattle. This and subsequent studies have suggested that LSDV may not replicate in infected insects, including *A. aegypti*, indicating only mechanical transmission [10, 11, 70]. However, the significance of *A. aegypti* in LSDV transmission in the field has been questioned due to this species displaying a preference to biting humans [70].

The biting insects found to have the greatest potential for LSDV transmission are *A. aegypti* mosquitoes and stable flies (*S. calcitrans*). Other *Stomoxys* spp. (*S. sitiens* and *S. indica*), horseflies (*Haematopota* spp.), biting midges (*Culicoides nubeculosus*), and ticks (*Rhipicephalus* (*Boophilus*) *decoloratus*, *Rhipicephalus appendiculatus*, *Rhipicephalus annulatus*, and *Amblyomma hebraeum*) also represent possible vectors [10, 11, 13, 42, 48, 49, 54–56, 64, 65, 71, 73, 79, 82]. Recent experimental studies have highlighted that *S. calcitrans* has a high reproduction ratio, is capable of retaining LSDV for at least 3 days, and is capable of mechanical transmission of LSDV to cattle [11, 48, 71, 79]. While there is only limited evidence for the biting midge *C. nubeculosus* as a potential vector for LSDV transmission, *Culicoides* spp. are reported to be common on cattle farms and therefore may pose a serious risk of LSDV transmission if they are proved to be efficient vectors of LSDV [11]. Various modes of LSDV transmission have been reported in different species of ticks: *R. decoloratus* (transstadial and transovarial), *R. appendiculatus* (mechanical, intrastadial, and transstadial), *R. annulatus* (transovarial), and *A. hebraeum* (mechanical, intrastadial, and transstadial) [7, 54–56, 73, 81, 82]. The detection of LSDV in the eggs of LSDV-infected *R. decoloratus* ticks is important because the eggs of this species are reported to be able to develop in soil and vegetation and thus present a risk of environmental contamination with LSDV-infected ticks [7, 81, 87]. Furthermore, experimental evidence has demonstrated that LSDV infection in female *R. decoloratus* ticks persisted following exposure to simulated winter conditions of 5°C at night and 20°C during the day for 2 months, with LSDV being detected in subsequently laid eggs [55]. This suggests that the virus may be able to over-winter in these tick species, which provides a possible means for LSDV to persist in the environment during colder months when biting insects are less active. In a field study of ticks collected from infected cattle during LSD outbreaks in Egypt and South Africa, LSDV was detected in a large proportion of different tick species, including *R. appendiculatus*, *A. hebraeum*, and *Rhipicephalus* (*Boophilus*) spp. [83]. These field and experimental findings provide evidence of ticks playing a potentially important role in the transmission of LSDV. In particular, the transovarial transmission of LSDV in certain tick species, combined with their short life cycle, indicates that they may act primarily as LSDV reservoirs rather than LSDV vectors [70].

Evidence suggests that approximately half of cattle infected with LSDV are asymptomatic, and that vectors are still capable of acquiring LSDV from these cattle [7]. Another study found that as few as four or five cattle in an LSDV infected herd of 100 head may show symptoms of LSD [64]. Due to lower viral titres, mechanical transmission of LSDV by vectors from asymptomatic cattle may be less efficient than that of clinically affected animals. One experimental study on mechanical transmission of LSDV from infected *A. aegypti* to cattle using one Holstein–Friesian steer and five Angus cross Jersey steers observed that one of the six cattle showed no clinical signs of LSD despite LSDV being detected in a blood sample from the animal; however, this study did not specify the breed of the sub-clinical steer [10]. Another experimental study, which tested mechanical transmission of LSDV from *A. aegypti*, *Culex quinquefasciatus*, *S. calcitrans*, and *C. nubeculosus* to eight male Holstein–Friesian cattle, classified five out of the eight cattle as being sub-clinical [11]. Both of these results may not accurately reflect the true incidence of sub-clinical LSDV infection in cattle due to their experimental nature.

Another potential factor influencing LSDV transmission is the fact that LSDV can survive in the skin lesions and scabs of cattle from 25 to 50 days to several months, making transmission still possible after other clinical signs of LSDV infection have subsided [7, 14, 44, 64]. Although direct contact is generally thought to be a less efficient means of LSDV transmission, exposure to nasal, lachrymal, and pharyngeal secretions may contribute to LSDV transmission, with LSDV able to survive up to 11 days in saliva [37, 77]. Additionally, LSDV has been reported to survive up to 42 days in fresh semen [39], and there are reports of LSDV being detected in semen excreted from bulls that have already recovered from the disease [39]. Experimental evidence has shown that LSDV transmission is possible during artificial insemination using LSDV-infected fresh semen [39].

### 3.3. Risk Factors for LSDV Exposure and Infection in Cattle in Endemic Countries

A number of factors can influence LSDV exposure and infection in cattle in endemic countries, including weather, physical environment, sociocultural factors, and inadequate biosecurity control interventions.

#### 3.3.1. Weather and Physical Environment

*(1) Land Surface Temperature*. Evidence has shown LSD to be more common in warmer months, when mean daily temperatures are higher [58, 77]. For example, Selim et al. [77] found a statistically significant increased risk of LSDV infection in Egypt during summer (odds ratio of 7.303 (95% confidence interval = 3.97–13.42)) compared to other seasons. The average mean surface air temperature in Egypt between 1991 and 2020 was approximately 30°C during the months of June–August [88]. A study in Russia found most LSD cases occurred during the summer months, when the mean daily temperature was 22.2°C [58]. This may be due to increased activity of vectors such as mosquitoes during summer months [7, 37, 65, 69, 77]. Conversely, a significant reduction in the transmission of LSD tends to occur during winter months, which is believed to be associated with a decrease in vector population [37, 61, 78]. Between May 2015 and August 2016, over 1,000 LSD outbreaks in cattle were reported in Türkiye and its neighbouring countries. The mean LSD spread rate for this period was 7.3 km/week; however, transmission of LSD decreased to an almost non-existent level during the winter months [61].

*(2) Rainfall*. Outbreaks of LSD have also occurred following periods of increased rainfall or monsoon weather, which is also likely to be associated with increased vector activity [37, 49, 65, 69, 78].

*(3) Migratory Birds and Associated Ticks*. A recent study found similarities in genomic sequences of LSDV isolates in cattle from Russia (Kinelsky) and Kazakhstan in 2019 and those from India (Ranchi and Odisha) in 2020–2021 [35]. A possible explanation of this long range and rapid movement of the virus is migratory birds infested with LSDV-infected ticks having migrated from Russia and Kazakhstan to India during the northern hemisphere winter and introducing LSDV to India [35]. This review only identified one study exploring the association between the movements of migratory birds and long-distance spread of LSDV, and therefore, further investigation is required to determine if this is a valid transmission pathway.

*(4) Wind Speed and Direction*. Limited evidence has also suggested that strong wind patterns may have enabled long-distance transportation of LSDV-infected vectors, such as stable flies, which are stronger fliers than mosquitoes. It is hypothesised that this process may have led to the introduction of LSDV-infected vectors from Egypt into nearby Israel, where an LSD outbreak was recorded in the village of Peduim in 1989 [51, 84]. One study identified five locations in Egypt, ranging in distance between 80 and 447 km to the outbreak area in Peduim, as possible points of origin for trajectories of LSDV-infected vectors caused by synoptic circulation pattern events. Of the five locations, Port Said (221 km) and Damietta (266 km) were associated with the highest number of events in the period leading up to the outbreak [51].

*(5) Proximity to Water Bodies*. Close proximity to water bodies, such as lakes, has been found to be associated with increased farm-level prevalence of LSD in Türkiye [78]. Seroprevalence of LSDV among cattle has also been found to be higher at farms in flood-prone and irrigated areas of Ethiopia [38, 43]. Both of these physical environments may be enhancing the activity and population of LSDV vectors.

*(6) Communal Grazing and Water Points*. A number of studies have found an increased risk of LSDV infection among cattle where communal grazing (grazing of cattle herds from different sources in the same area) and communal water points occur [1, 27, 37, 41, 65, 66, 68, 69, 77], with one study finding odds ratios of 1.546 (95% confidence interval = 0.91–2.60) and 3.283 (95% confidence interval = 2.11–5.09) for these associations, respectively [77]. Another study found odds ratios of 4.1 (95% confidence interval = 2.02–6.18) and 8.5 (95% confidence interval = 6.0–11.0), respectively, for the same risk factor associations [41].

#### 3.3.2. Sociocultural Factors (including Economic and Political Factors)

*(1) Seasonal Festivities*. Traditional events such as the Eid festival have been associated with increased movement of livestock both within and between the countries of Pakistan and Türkiye [5, 7, 45, 50]. This resulted in a major LSD outbreak in southern Punjab province following the Eid festival in 2022, when there was a spike in animals being brought from Sindh province to Punjab province for slaughter [45, 50].

*(2) Political Factors*. Political unrest and conflicts in Syria and Iraq have resulted in a significant number of refugees and their animals fleeing to neighbouring countries, including Türkiye and Jordan, and these events may have played a large role in a number of LSD outbreaks between 2012 and 2014 in neighbouring Israel, Lebanon, Jordan, Türkiye, Iraq, and Iran. Furthermore, Türkiye and Jordan have been put under financial pressure in supporting the refugees, resulting in them being unable to provide adequate veterinary care or infrastructure [7].

*(3) Farmers' Knowledge, Attitudes, and Practices*. Farmers' lack of knowledge on LSD and its transmission has been identified as a potential factor associated with LSD outbreaks in both LSD endemic and countries with recent LSD incursions [4, 8, 30, 47, 53]. A recent study highlighted that most farm owners in China lack knowledge or awareness of LSD, which may be contributing to under-reporting of suspected LSD cases to the government, in addition to inadequate disease control strategies such as proper disposal of infected animals [4]. Another study found that farmers in Nigeria often sell their infected animals at livestock markets or slaughter them at low prices to support their livelihood. The authors of the study suggest that education of farmers on LSD transmission may help farmers make more informed decisions when purchasing animals [53]. The selling of infected animals in low-resource countries may not be solely due to lack of education, but rather out of economic necessity due to lack of compensation for reporting infected animals to authorities, whereby the affected animals may be confiscated [89].

#### 3.3.3. Inadequate Biosecurity Control Interventions

*(1) Quarantine*. A study in Nigeria has shown that the purchase of replacement animals takes place at live cattle markets, making them a hub for spreading LSD [53]. Mixing of cattle from different herds can lead to close contact and providing increased opportunity for transmission of LSD via vectors carried by the cattle or direct contact between uninfected and infected cattle [37, 77]. Despite government-supported LSD control interventions being in place in Albania, LSD has not been eliminated from the country. A study identified that there are insufficient biosecurity measures in place to prevent inter-herd mixing in Albania, which is compounded by a lack of clear boundaries between farms in most cases [52]. A study of farmers in Ethiopia identified that insufficient quarantine of newly purchased cattle before introducing them to the herd may be linked to higher occurrence of LSDV infection [43].

*(2) Vaccination*. Israel was able to control an LSD outbreak in 2012–2013 by deploying a mass vaccination campaign, with no subsequent cases of LSD reported in the following years. In contrast to this, when Türkiye introduced a similar country-wide vaccination programme to control an LSD outbreak in 2012, it was unsuccessful in controlling LSD. This outcome suggests that in addition to vaccination, other interventions may be required in some regions to control the spread of LSDV infection [13].

Another study highlighted varying effectiveness of LSD control interventions in China, including restriction of animal movement and vaccination. These measures were effective in Xinjiang province following reporting of LSD in July/August 2019; however, LSD was not controlled successfully when a similar approach was taken in Fujian province when it was detected there in June 2020. Furthermore, LSD continued to spread to a number of other neighbouring provinces and to Taiwan, less than 200 km off the coast of Fujian province, during the following month. This study suggested this difference in effectiveness may have been due to lack of awareness of LSD and control interventions among veterinarians and farmers in Fujian province, as well as a low uptake of vaccinations [57]. It is believed that insufficient infrastructure for detecting LSD in a timely manner and public awareness of LSD are vital aspects of controlling the spread of LSD [6].

Mass vaccination using live-attenuated vaccines has been used with varying degrees of success in several countries to control LSD outbreaks, as recently reviewed by Akther et al. [90]. The vaccines used in these control programmes have been based on exogenous LSDV strains (e.g., the Neethling strain and its derivatives), endogenous LSDV strains, and various strains of sheep poxvirus and goat poxvirus [90]. LSD vaccines have; however, been reported to sometimes cause side effects, including lumps that are smaller and fewer than seen in cattle infected with LSD, fever, and a reduction in milk production [91]. The use of live-attenuated vaccines in LSD-free countries presents a potential problem in that it may lead to the continuation of international trade restrictions as differentiating infected from vaccinated animal (DIVA) principles cannot be applied. As a result of antigenic conservation, sheep and goat poxvirus-derived vaccines have also been used for controlling LSDV, though their effectiveness in cattle has been questioned [92]. The close antigen similarity between sheep and goat poxvirus strains to LSDV prevents the application of DIVA principles [93], thus making them unsuitable for use in LSD eradication programmes or control of LSD outbreaks. Moreover, in some countries like Australia, these viruses are also classified as exotic viruses, making their use impractical from a freedom of disease perspective. Development of inactivated or recombinant vaccines may allow for LSD-free countries to vaccinate cattle populations without facing prolonged international trade restrictions [7].

### 3.4. Risk Factors Influencing Probability of Introduction (Illegal Processes)/Importation (Legal Processes) and Probability of Detection of Infected Cattle from Endemic Countries to LSD-Free Countries

*(1) Probability of Introduction*. Live animal movement by both legal and illegal means provides opportunities for the transmission of LSDV. A recent study suggested that this was more likely to contribute to the wide geographical spread of LSDV infection, than mechanical transmission via vectors [7]. Most LSD-free countries have implemented WOAH recommendations on preventing trans-boundary spread of LSD; however, these interventions have become difficult to enforce in some instances, including on the European Union (EU) border [36]. The emergence of LSD in China in 2019 can be partly explained by cattle movement from neighbouring LSD endemic countries into China [4]. A study exploring the risk of introduction of LSD into the United Kingdom (UK) through legal importation of skins, hides, and wool from the EU found that the probability of LSD being introduced by a single hide/skin/wool bale from an EU member state where LSD outbreaks are ongoing was low [40].

*(2) Probability of Detection*. While EU member state reference laboratories are able to detect LSDV, it is up to farmers and veterinarians to first recognise any clinical signs of LSD in newly imported cattle in the field before their infection status can be confirmed in a laboratory. However, this can be challenging during the early stages of infection because LSD clinical signs can be confused with clinical signs of other diseases and cattle may not be monitored daily. The fact that many LSDV infections are asymptomatic, combined with the challenges associated with detecting and diagnosing LSDV infection in a timely matter in the field, makes it difficult to control transmission of LSDV to vectors and cattle. These factors can render any culling of LSD-infected cattle and those they have been in contact with ineffective as the virus may have already dispersed from the point of detection.

### 3.5. Surveillance Strategies That Are Being Implemented in LSD-Free Countries

A wide range of LSD control strategies have been implemented in LSD endemic countries, including vaccination, restriction of cattle movement, culling of infected and exposed cattle, and vector control [13, 37].

Registration of cattle movement, as part of surveillance and monitoring, has been implemented in a number of LSD-free countries. In Switzerland, it is a legal requirement for all premises keeping cattle to be registered, as well as for all cattle movement and culling to be reported within 3 working days to the Swiss cattle movement database (Tierverkehrsdatenbank, TVD). This timely monitoring of cattle movement allows for rapid contact tracing of cattle, especially when potentially infected cattle are still in the incubation period [44]. Ukraine also has strict laws in place for LSD prevention, whereby the importation of cattle and biomaterials, such as hides and semen, are only allowed from LSD-free countries, where surveillance and monitoring systems are in place. While a strong national LSD prevention strategy is in place, it is not clear whether current farm-level biosecurity measures and farmers' awareness of LSDV infection are sufficient to respond to LSDV incursions in their herds [39].

The UK has a comprehensive LSD control strategy, which sets out clear and specific actions to be taken in the event of a suspected or confirmed case of LSD in the country. The control strategy outlines the steps to be taken to report and diagnose/confirm suspected LSDV infection, response activities including culling, cleansing and disinfection (including vector control at infected premises), and contact tracing, establishment of disease control zones for movement and trade restriction, monitoring and surveillance of live animals, possible deployment of vaccination, a recovery plan for the control zones, and the process for resuming international trade and regaining disease-free status. This document provides advice for governments, industries, and individuals involved with LSD susceptible animals [91].

### 3.6. Epidemiological Approaches for LSDV Decision Support

Fourteen articles that presented a number of different epidemiological modelling approaches were identified [5, 11, 31, 32, 34, 42, 58, 59, 62, 63, 67, 72, 75, 76]. These studies have applied epidemiological modelling to various aspects of LSDV infection and transmission to support decision making in designing and implementing LSD control strategies.

#### 3.6.1. LSDV Transmission Models

A limited number of LSDV transmission models have been developed for LSD-infected countries to estimate the reproduction ratio (*R*_0_) of LSDV transmission between uninfected and infected cattle, transmission of LSDV by vectors, and retention of LSDV in vectors.

*(1) Cattle to Cattle Transmission*. A susceptible-exposed-infectious-recovered (SEIR) epidemiological model was applied to model LSDV transmission between cattle in Türkiye to determine production losses during an LSD outbreak. This study highlighted that detection of LSD in cattle during the incubation period can reduce economic loss associated with an LSD outbreak [5]. Another study compared the *R*_0_ for three different routes of LSDV transmission in a cattle herd in Israel: indirect contact between the groups within a herd, direct contact or contact via common drinking water within the groups, and transmission by contact during milking. Of these, only indirect transmission produced a high *R*_0_ value (*R*_0_ = 15.7), suggesting that flying biting insects may have been the primary mode of LSDV transmission in the herd [59].

*(2) Farm to Farm Transmission*. A susceptible-infectious-recovered (SIR) epidemiological model was applied to estimate LSDV transmission between cattle in mixed livestock herds, which were known to mix at shared pastures and watering points, and intensive commercial herds, which were isolated from other herds, in Ethiopia, and found a similar R_0_ between these two types of herds [63].

*(3) Vector Transmission Efficiency*. A study of five different species of biting insects: the stable fly (*S. calcitrans*), the biting midge (*C. nubeculosus*), and three mosquito species (*A. aegypti*, *Anopheles stephensi*, and *C. quinquefasciatus*) analysed the reproduction ratio for LSDV transmission for these species, based on data on mechanical transmission of LSDV taken from available literature. The study estimated *S. calcitrans* and *A. aegypti* as having high reproduction ratios, with the other three species likely to be inefficient vectors of LSDV [42]. An experimental study on LSDV vector transmission efficiency found *S. calcitrans* to have a very high reproduction ratio (*R*_0_ = 19.09; 95% credible interval = 2.73–57.03), with *C. nubeculosus* (*R*_0_ = 7.09; 95% credible interval = 0.24–37.10) and *A. aegypti* (*R*_0_ = 2.41; 95% credible interval = 0.50–5.22) also suggested as being potentially efficient transmitters of LSDV [11]. The estimates for *R*_0_ from these two studies both incorporated three vector life history parameters: biting rate (time interval between blood meals), vector to host ratio, and vector mortality rate. The importance of these vector life history parameters is evidenced by the wide 95% credible intervals provided with the experimental estimated values for *R*_0_ produced by Sanz–Bernardo et al. [11]. Both studies assumed constant values for these vector life history parameters; however, these are likely to vary depending on environmental factors, such as climate and habitat suitability, which are influenced by geography and seasonality. This means that *R*_0_ is also likely to vary over space and time [11, 42].

*(4) Cattle to Vector Transmission*. A recent experimental study for the first time quantified the difference in probability of uptake of LSDV by four biting insect vectors (*A. aegypti*, *Cx. quinquefasciatus*, *C. nubeculosus*, and *S. calcitrans*) that were exposed to sub-clinical or clinically infected cattle. A marked difference was found in the probability of vectors acquiring LSDV from sub-clinical animals compared to clinical animals, with vectors being 97% less likely to acquire LSDV from feeding on a sub-clinical animal than on a clinical animal, with estimated probabilities of transmission from cattle to insect of 0.006 and 0.22, respectively. However, this ratio of probability of transmission was not found to differ between the four species of biting insect tested. This study also highlighted that the probability of transmission from cattle to insects was low during the pre-clinical stage of the disease, during which viremia is relatively low and skin lesions have yet to appear [11].

#### 3.6.2. Modelling Studies in LSDV-Infected Countries

*(1) Time-Series Forecast Models*. *(i) National Level*. A study of LSD outbreaks in Ethiopia between 2000 and 2015 developed an autoregressive integrated moving average (ARIMA) model, which identified seasonality in the number of outbreaks each month each year. Importantly, the model was able to forecast the months with the highest number of LSD outbreaks between 2016 and 2018. Furthermore, the Spearman rank test was performed to test for any lag effect between the monthly number of LSD outbreaks and monthly rainfall, with a statistically significant correlation found at 3 months, indicating LSD outbreaks were most common following increased rainfall occurring 3 months earlier [62].

*(ii) Global Level*. Another study applied ARIMA and neural network autoregressive (NNAR) models to predict future trends in LSD outbreaks across the world in 2023–2024 using the numbers of LSD reports in Africa, Europe, and Asia recorded on the publicly available WOAH database from 2005 to 2022. It predicted that the number of LSD outbreaks would remain stable in Europe but increase in Africa and Asia [31].

*(2) Cluster Analysis*. A recent study of the first LSD outbreak in Thailand in Roi Et province in 2021 applied space–time permutation (STP) and Poisson space–time (Poisson ST) models to detect areas of high LSD incidence in the country. The cluster size of 17.7 km identified by the latter of these models validated the outbreak control programme implemented by the Department of Livestock Development (DLD), where authorities had been advised to prioritise disease control in the surrounding areas falling within a 30 km radius of farms where an LSD outbreak is detected [34]. In contrast to this, another study of the LSD outbreaks in Khon Kaen province in Thailand in 2021–2022 found much smaller clusters in the range of 1.5–4.5 km using space–time permutation, Poisson, and Bernoulli models [72]. The findings of these two studies suggest that the size of clusters of LSD outbreaks may vary between different geographical areas and may also vary at different stages in the spread of LSD in a country [34, 72]. Further studies could compare the sizes of clusters of LSD outbreaks in the different regions of Thailand to explore possible reasons for the differences in their sizes and whether the stage of the spread of LSD in the country plays any role in cluster size.

A study of LSD outbreaks in Uganda between 2002 and 2016 used spatial, temporal, and space–time scan statistics to identify LSD outbreak clusters. The study found seven statistically significant purely spatial clusters of LSD outbreaks in different locations within Uganda, with some occurring in individual districts and others spanning multiple districts with radii ranging from 25.35 to 55.57 km. A single space–time cluster was identified with a radius of 168.37 km and spanning 4 years from 2002 to 2005. The space–time cluster primarily spanned a majority of the central and eastern regions of the country. Outbreaks in these two regions made up 68% (790/1161) of LSD outbreaks reported between 2002 and 2016 in Uganda. Furthermore, LSD outbreaks in the central and eastern regions were found to be less seasonal and had significantly lower mortality than the other regions of Uganda, suggesting possible geographical differences in disease epidemiology [67]. These studies in Thailand and Uganda highlight the differences in clustering of LSD outbreaks that can vary between geographical areas within a country.

*(3) Spatio-Temporal Modelling and Risk Mapping*. *(i) National Level*. A maximum entropy ecological niche model was developed to quantify the probability of LSD outbreaks in Iran based on data from 2012 to 2016. The model evaluated the influences of environmental variables on the spatial distribution of the risk of an LSD outbreak occurring. The model identified a probability of LSD outbreak of 60% or greater in areas where precipitation in the coldest season is between 140 and 160 mm, mean temperature during the wettest season is between −1 and 6°C, and precipitation in the wettest season is between 58 and 62 mm [32].

*(ii) Regional Level*. A study of LSD outbreaks in Iraq, Iran, Türkiye, Kazakhstan, and several countries in Eastern Europe, including the Russian Federation and the countries of the Balkans during 2014–2016, developed a Bayesian hierarchical model, which incorporated environmental factors and the spatio-temporal distribution of LSD outbreaks. The multivariable model found statistically significant associations between the risk of an LSD outbreak occurring and maximum temperature, precipitation, and wind speed. These results were complemented by developing a map of environmental suitability and the changing spatio-temporal distribution. Such maps provide a useful tool for identifying spatial heterogeneity rates of LSD outbreaks in environmentally heterogeneous regions [58].

#### 3.6.3. Modelling of Transboundary Transmission into LSD-Free Countries

*(1) Risk from Imported Cattle*. A study in France developed a stochastic quantitative import risk analysis (QIRA) model to evaluate the risk of LSD being introduced into the country through imported cattle from the Balkans [76]. The model estimated the probability for this risk for the scenarios of cattle being imported for breeding or fattening or for slaughter, as well as the yearly probability of LSDV-infected imported cattle transmitting the virus to native cattle for these two scenarios. These probabilities were modelled by applying data on the number of cattle imported from at-risk countries into France and a number of probability parameters on probability of importing LSDV-infected animals, which were based on a combination of experimental data, field data, literature, and expert opinion. The parameters for cattle importation numbers were the “number of cattle imported per year and the number of batches imported per year,” both of which were determined for farms and slaughterhouses separately. Additionally, these parameters were used to calculate secondary parameters: “average number of imported cattle heads per batch entering farms” and “average number of imported cattle heads per batch entering slaughterhouses.” The probability parameters included in the model were as follows: “probability of importing cattle from an at-risk area that may become infected with LSDV before its detection,” “probability of importing animals from an infected farm from this area,” “probability of infection for a given animal from this farm,” “probability that an infected animal is infectious,” and “probability that an infected and infectious animal transmits the infection to native animals in France.” The last of these probability parameters was determined for three different scenarios: farm as a destination in summer months, farm as a destination in winter months, and slaughterhouse as a destination. The model estimated that the probability of imported cattle coming from an infected farm and transmitting LSDV to native cattle was three times higher in summer months than in winter months [76].

*(2) Risk from Introduction of Infected Flies*. A similar study applied a QIRA model to quantify the risk of LSD being introduced into France by stable flies (*S. calcitrans*) found in animal trucks from at-risk countries (those of the Balkans and neighbouring countries). The model estimated the probability of native cattle being infected with LSDV by stable flies that entered the country for the three scenarios of stable flies travelling with cattle going to farms, with cattle going to slaughterhouses, or with horses. These probabilities were modelled by applying data on the number of cattle and horses imported from these at-risk countries into France by truck, the number of stable flies travelling with these imported animals, and a number of probability parameters. The probability parameters were as follows: “probability of importing cattle from an at-risk area that can become infected with LSDV before its detection,” “probability that trucks come from an infected farm located in the at-risk area,” “probability of a animal being infected without clinical signs in the farm,” “probability of the virus surviving in the Stomoxys,” “probability of Stomoxys surviving during transport (2 ± 3 days),” “probability that LSDV is transmitted at the destination in the event of a truck transporting cattle to a farm,” “probability that LSDV is transmitted at the destination in the event of a truck transporting cattle to a slaughterhouse,” “probability that LSDV is transmitted at the destination in the event of a truck transporting horses,” “probability that horses come from a mixed farm (with cattle) or that a cattle farm is in the vicinity of the stables,” and “probability that horses go to a mixed farm (with cattle) or that a cattle farm is in the vicinity of the stables.” The model identified a higher probability of *S. calcitrans* entering farms in France from vehicles transporting cattle from at-risk countries to farms (median probability = 8.99 × 10^−4^; 95% confidence interval = 6 × 10^−5^−5.93 × 10^−3^), compared to *S. calcitrans* entering via trucks transporting horses (median probability = 5.82 × 10^−9^; 95% confidence interval = 5.0 × 10^−10^−3.95 × 10^−8^) or trucks taking cattle to slaughterhouses (median probability = 4.27 × 10^−6^; 95% confidence interval = 2.0 × 10^−7^−3.73 × 10^−5^) [75].

## 4. Discussion

This scoping literature review identified a total of 68 articles reporting evidence on risk factors for exposure and spread and epidemiological modelling approaches to support disease control decision-making pertaining to LSD.

### 4.1. Risk Factors for LSDV Exposure and Spread

A number of common themes were identified in the 47 reviewed articles relating to risk factors for LSDV exposure and infection in cattle in endemic countries. First, environmental factors shown to increase the risk of LSDV infection in cattle included high average daytime temperature, high rainfall, and close proximity to water bodies. These weather characteristics and physical environmental properties correspond to the ideal conditions for vector activity and cattle exposure to vectors. Furthermore, stable flies are known to be able to travel long distances (up to 29 km in laboratory tests [84]), with this distance being potentially further extended by strong wind patterns, and marked flies having been recaught up to 83 km from their point of release [84]. However, there is no direct evidence of LSDV transmission by stable flies that have travelled long distances [37, 38, 43, 51, 61, 65, 69, 77, 78, 84]. Second, in LSD endemic countries, the practices of communal grazing and communal water points are common. This mixing of herds presents the possibility of exposure to LSDV both via vector transmission and exposure to nasal, lachrymal, and pharyngeal secretions [1, 27, 37, 41, 65, 66, 68, 69, 77]. Third, awareness among farmers regarding LSDV transmission, along with the degree to which farm-level biosecurity practices are implemented throughout the country, is also reported as important risk factors in some LSD endemic countries. This may in turn be hampering efforts to control the spread of LSDV infection in these countries [4, 8, 30, 47, 53]. Fourth, environmental contamination by LSDV-infected tick eggs in soil and vegetation has been reported, and experimental evidence suggests that LSDV infection can persist in female *R. decoloratus* ticks under simulated winter conditions; however, there is limited evidence of how important a role ticks play in the transmission of LSDV [7, 55]. Fifth, LSDV transmission through cattle movement occurs as a result of illegal trade, as well as large-scale movement of animals including cattle following cultural events and migration of refugees due to political unrest. Cattle movement is thought to be the main factor in the large geographical spread of LSDV [5, 7, 45, 50]. Finally, while vaccination has been effective in controlling the spread of LSDV in endemic countries, the only currently available vaccines are live-attenuated vaccines, which cannot be used as a preventative measure in LSD-free countries without the risk of international trade restrictions remaining [7, 13, 57, 91]. Additionally, there have been reports of vaccine-like recombinant strains of LSDV in Asia, with studies suggesting that these strains may have risen from the use of poorly manufactured LSDV vaccines [94]. These findings highlight the need for control measures in the deployment of LSDV vaccines.

The inclusion and exclusion criteria applied to the literature search used in our review were devised to extract key data that would assist in the development of spatial epidemiological disease modelling; however, these may have limited the scope of risk factors identified. Broader inclusion criteria may have helped identify additional risk factors, which may have helped in describing the effects of the risk factors identified in this review.

### 4.2. Epidemiological Approaches for Modelling LSDV Exposure and Spread

To date, a limited number of studies have presented mathematical models estimating the potential risk of LSDV transmission from possible vectors, with LSDV infection in cattle by stable flies and *A. aegypti* mosquitoes in particular showing a potentially high reproduction ratio. All modelling studies related to spatio-temporal distribution of LSDV infection risk, including time-series forecast models, cluster analysis, and spatio-temporal modelling and risk mapping, were based on data from LSDV endemic countries [5, 11, 31, 32, 34, 42, 58, 59, 62, 63, 67, 72]. These studies' findings confirmed the important climate risk factors of higher temperature and rainfall, as well as identified geographical areas most at risk of LSDV outbreaks occurring, along with when they are most likely to occur. The only studies from LSD-free countries describing epidemiological approaches for modelling risk of LSDV importation were from France, and their focus was on probabilities of LSDV entering the country via different methods, including importation of infected live cattle and infected vectors being moved on vehicles transporting livestock into the country [75, 76]. The literature review did not identify any studies applying multi-criteria decision analysis modelling to look at the suitability of LSD-free countries for LSDV incursions, such as distribution of cattle population and climate and habitat factors necessary for the establishment of infected vectors.

### 4.3. Implications of Review Findings to LSDV Epidemiological Research in Australia

Based on the reviewed literature, the Australian landscape presents a wide range of biotic and abiotic optimal conditions for LSDV incursion and spread. The populations and species of vectors present in different parts of Australia may vary, and therefore, understanding the distribution of competent vectors in Australia will be valuable in identifying areas at potentially high risk of LSDV transmission in the event of introduction of LSD into Australia. In addition, there is considerable arthropod-borne viral (arboviral) activity in northern Australia that is monitored through the National Arbovirus Monitoring Program (NAMP) for livestock viruses of interest to key export markets, such as bluetongue virus, Akabane, and bovine ephemeral fever virus. It would seem prudent to expand this programme to include LSDV surveillance in this well-established system to increase the likelihood of early detection of any incursion by the virus [95].

Available literature describes *R. decoloratus* as having potential for transovarial transmission of LSDV and being able to survive winter conditions. While its short life cycle may limit its ability for rapid spread of LSDV, this species may act as an LSDV reservoir [55, 70]. The Australian cattle tick, *R. australis*, is reported to be closely related to *R. decoloratus* [18, 96, 97]. While members of the *Rhipicephalus* genus, including *R. australis* and *R. decoloratus*, are known vectors of other important livestock pathogens such as *Babesia bovis*, *Babesia bigemina*, *Anaplasma marginale*, and *Theileria parva* [98], recent studies have reported a much wider repertoire of viruses being detected in ticks of potential importance to mammalian host species [98, 99]. Future studies are warranted to investigate the ability of the Australian cattle tick to act as a vector for mechanical transmission of LSDV or as a reservoir of LSDV. These studies should also evaluate the capacity of *R. australis* to vertically transmit LSDV, as has been demonstrated for *R. decoloratus* [81]. Vertical transmission would be the most important mode of *R. australis*-mediated transmission of LSDV, as it completes its life cycle on a single host, thus negating direct horizontal host transmission.

Another potential source of LSDV entering Australia is via migratory birds through the East Asian–Australian migratory flyway. The bird species using this flyway have been of interest with respect to avian influenza [100]. Critical points of difference between avian influenza and LSDV are that birds are considered to be the natural reservoir of influenza viruses and they are not susceptible to LSDV infection. As such, for migratory birds to play a role in an LSDV incursion into Australia, it would need to be via carriage of a suitable vector, such as ticks. As described previously, bird–tick interactions have been proposed as a possible source of long-range movement of LSDV [35]. While this possibility cannot be excluded as a potential source of LSDV incursion into Australia, it is considered unlikely as there is no overlap of tick species between Australia and countries such as Indonesia, with no evidence of new tick species entering and becoming established in Australia in recent history [101].

Previous LSD outbreaks in Israel were thought to have been due to the long-range dispersal of LSDV-infected vectors from Egypt being carried by strong wind patterns [51]. A similar precedent exists in Australia, where Culicoides biting midges infected with bluetongue virus are thought to have been blown into the top end of the Northern Territory from overseas by warm humid winds. These winds can reportedly carry insects several hundred kilometres [102]. This suggests that LSDV-infected vectors could potentially also be transported into the north of Australia from neighbouring countries via wind currents.

Intrinsically, linked to the availability of a suitable vector(s) for LSDV in Australia is host susceptibility. Given the proximity of the northern Australia cattle production systems to countries where LSDV is currently circulating, it is reasonable to conclude that it is the most likely incursion point from non-anthropologic means. There is likely to be variable susceptibility to LSDV among Australian cattle herds. This variability may be driven by the gradient of *Bos indicus* dominant genetics to *B. taurus* dominant genetics from across northern Australia down the eastern states through to the southern production systems [103]. While this genetic gradient is largely driven by the capacity of *B. indicus* genetics to be productive in the harsher environments of tropical and sub-tropical Australia, it may also make the detection of LSDV incursions problematic. It is widely accepted that *B. indicus* breeds have increased innate resilience to LSDV infections compared to European *B. taurus* breeds [104, 105]. Recently, Sudhakar et al. [106] reported on LSDV outbreaks in India with overall 7.10% morbidity (range: 0.75%–38.34%) and 0% mortality in cattle (*n* = 2,539) from five locations. The cattle (*n* = 133) from the location with the highest morbidity, an organised farm, had a mixed breed population consisting of *B. indicus*, *B. taurus*, and *B. indicus* × *B. taurus*, though the specific breakdown of the numbers of cases for each breed was not reported [106]. As noted by the authors, while breed was likely to be an important factor in the variable morbidity rates and lack of mortality, the virulence of the LSDV strains at each location may have also contributed to these results [106]. It is also worth noting that some indigenous *B. taurus* breeds (e.g., Egyptian Baladi cattle) are reported to have reduced LSDV susceptibility compared to European *B. taurus* breeds [77].

Consequently, there could be two outcomes in the event of an LSDV incursion into this region. First, as a result of the *B. indicus* genetics in this production system, these cattle may naturally resist a low-level outbreak. While this might be considered a desirable outcome from a trade perspective, it could mean potential incursion points are not identified in a timely manner to allow the implementation of appropriate management controls. Second, the natural resistance of these breeds may suppress the expression of clinical signs, making the timely identification of affected animals more problematic in the early stages of infection, potentially exacerbating the outbreak prior to its detection. Although as described previously, pre-clinical and sub-clinical infections may be less likely to facilitate vector-borne transmission [11]. Potentially further confounding these outcomes are feral populations of buffalo and, to a lesser extent, feral cattle, in northern Australia that are likely to be susceptible to LSDV. By the very nature of these herds, early detection of LSDV, let alone control or eradication, is likely to be highly problematic.

With the recent detection of LSD in countries neighbouring Australia to its north, such as in Indonesia, the risk of importation of LSD into northern Australia is considered non-negligible. The existing strict biosecurity measures in place effectively reduce the risk of importation of LSD via infected animals or related products, with restrictions on the countries of origin. The Australian Government Department of Agriculture, Fisheries and Forestry regulates the importation of reproductive materials, such as bovine semen, with strict rules and procedures in place to prevent the introduction of diseases into Australia's animal populations [107]. However, the risk of importation via infected insects entering through international ports or travelling over sea into northern Australia is considered high. The Australian Government has strict biosecurity measures in place at all international ports to minimise the risk of infected animals or materials entering the country; however, it also needs to ensure a high level of awareness of LSDV is maintained among farmers, veterinarians, and other related individuals. Disinsection is conducted at international ports; however, it is suggested that development of insecticide resistance by vectors may increase the risk of importation of LSDV into Australia via infected vectors. Australia has developed a nationally agreed policy for controlling LSD in a timely manner in the event of a suspected or confirmed case of LSD in the country [18]. Australia's animal health surveillance system enables the early detection of LSD. In particular, the Northern Australia Quarantine Strategy (NAQS) has implemented routine testing of both domestic and feral animals for LSD in Northern Territory, Queensland (including the Torres Strait), and Western Australia [17]. Biosecurity measures, including both preventing LSDV entering the country and controlling LSDV infections in the event of an incursion, are important in preventing and mitigating the potential economic impact on Australia's cattle industries. An example of a disease affecting cattle and impacting national economies is a foot and mouth disease (FMD), where outbreaks in other countries have impacted dairy production in cattle, resulting in economic losses due to restrictions on exports of dairy products [108]. A previous study modelled the potential economic impacts of FMD entering Australia and estimated that there would be significant losses, in terms of both national cattle production and export earnings, in the event of an outbreak in the country [95]. In the first year of the outbreak, the economic impact was estimated to be 0.6% of gross domestic product (valued at AUS$3.5 billion in 1999–2000) and 0.8% drop in employment, highlighting the need for government and industry cooperation in the prevention and control of FMD [109]. More recent modelling has estimated the total direct economic impact of an FMD outbreak in Australia at AU$80 billion (2020–2021 AUD) over 10 years [110].

The Australian control strategy provides more specific recommendations on controlling the spread of LSD, such as vector management, with an emphasis on the need for responses that are tailored to the various environmental conditions and seasons in Australia [18]. In addition to this control strategy, Australia has set out an action plan to actively take steps to strengthen preparedness for a potential introduction of LSD into the country. The plan has set a number of actions to achieve this, under the objectives of developing international engagement, border biosecurity and trade, diagnostic capability and capacity, surveillance, preparedness and response, awareness and communications, research and innovation, and recovery. As part of this preparedness plan, Australia is supporting Indonesia's LSD outbreak response and is helping to improve technical and diagnostic capability and surveillance [111].

The most common practice for control of LSDV in endemic countries has been extensive vaccination programmes in cattle, using live-attenuated vaccines. However, this is not a practical preventative measure in LSD-free countries such as Australia as DIVA principles cannot be used to distinguish vaccinated animals from infected animals. As such, the use of live-attenuated vaccines could prolong the potential economic impact of an outbreak through continuation of international trade restrictions.

However, Australia's action plan sets out priorities aimed to enhance Australia's ability to prevent, detect, prepare for, and respond to a possible LSDV incursion. Activities in the plan include developing a national cattle vaccination strategy and modelling systems for LSD. The plan highlights the importance of being able to map and model cattle populations and movements through resources such as the Australian National Livestock Identification System (NLIS, a lifetime cattle movement database) to support LSD surveillance and preparedness activities [111]. The capacity of the NLIS database to inform the effective tracing of cattle movement and mixing with respect to modelling disease risk has previously been demonstrated [112].

Recently, qualitative and quantitative risk assessments of four specific unregulated pathways for entry of and exposure of LSDV in Australia were performed: (1) wind-borne dispersal of arthropod vectors, (2) commercial vessels carrying hitchhiker arthropod vectors (excluding live export vessels), (3) returning live export vessels carrying hitchhiker arthropod vectors, and (4) Torres Strait Treaty movements carrying hitchhiker arthropod vectors [22, 23]. The risk assessments developed a parameterised model, based on evidence collected via consultation with experts and a literature review, to evaluate three different scenarios: (1) at least 30–50 insects are necessary for successful vector-to-bovine transmission of LSDV, (2) several (i.e., 3–5) vectors are necessary for transmission, and (3) a single insect is sufficient for transmission, and estimated incursion risk using a two-dimensional Monte Carlo simulation. The estimated risk for the first two scenarios was negligible to very low, with a substantially increased risk in the third scenario; however, the risk assessment noted that this third scenario has not been achieved experimentally. The highest risk pathway was found to be the wind-borne dispersal of infectious vectors, with the highest likelihood of LSDV incursion associated with areas within the Northern Australian Quarantine Strategy (NAQS) risk zones, including the Tiwi Islands and regions east of Darwin, extending to and including the Cobourg Peninsula. These findings support those of studies, which suggested that LSDV-infected stable flies from Egypt may have introduced LSDV into Israel as a result of strong wind patterns [51, 84].

Importantly, there were significant uncertainties in the model parameters due to lack of data availability (e.g., numbers of herds symptomatically infected with LSDV overseas), lack of consensus among experts on probability estimates (e.g., infectious vectors being able to successfully travel via wind), and the use of proxy data (e.g., urbanisation index as a proxy for the likelihood that an infectious vector would travel to a seaport in the country of origin) [22]. Consequently, the risk assessments emphasise that the results do not accurately represent the true risk of LSDV incursion into Australia. Future experimental studies could be designed to more accurately measure these parameters.

The risk assessments also suggest that the risk of LSDV incursion into Australia may be increasing due to climate change. In particular, this is thought to have led to an increase in the frequency of suitable meteorological conditions for wind-borne vector dispersal and changes to the Australian environment that have expanded the areas favourable for LSDV vector activity over recent years [23]. Presently, modelling has only explored the risk of an LSDV incursion into Australia; however, these need to be supplemented by models that incorporate such environmental changes to identify the geographical areas with the highest potential for the spread of LSD in the Australian cattle population following an LSDV incursion in order to support risk-based surveillance of LSD in Australia.

The Australian Government National LSD Action Plan highlights the need for developing modelling tools that can quickly assess the changing risk profile of LSD. Furthermore, the action plan draws attention to the Australian Animal DISease (AADIS) model, which can simulate the spread and control of animal disease in Australia, and that there is a need for the development of such tools to predict appropriate controls for LSD [111].

## 5. Conclusions

Some commonalities were found in the risk factors associated with LSDV spread among geographically varying LSD endemic countries, such as increased rainfall, high average daytime temperature, and the proximity of cattle to water bodies. These were all associated with increased vector activity and cattle exposure to vectors. While certain species of mosquitoes, flies, midges, and ticks have been implicated in LSDV outbreaks, evidence of their LSDV transmission efficiency has only been explored experimentally, with high reproduction ratios observed in *A. aegypti* mosquitoes and stable flies (*S. calcitrans*). LSDV transmission by flying vectors, such as stable flies, travelling long distances aided by strong winds, is also thought to be possible. Several studies suggest that the most likely route of wide geographical spread of LSD is cattle movement across countries' land borders.

This review observed a lack of studies modelling risk of LSDV incursion in LSD-free countries. The identified literature only modelled risk of LSD introduction through specific entry pathways. Furthermore, no literature describing the models of LSDV spread following introduction of the virus in LSD-free countries was identified.

Risk assessments of potential LSDV incursions into Australia via various pathways have been conducted; however, gaps remain in accurately modelling the true risk of LSDV incursion. Additionally, there are no models of how LSDV could spread between cattle in Australia following an LSDV incursion. The findings from studies on LSDV outbreaks in other countries may provide a useful basis for developing models of LSDV incursion and spread in Australia; however, these will need to be adapted to Australia's unique geographic and environmental profile, as well as its different livestock and vector populations. In addition to these, modelling will also need to consider a wide range of risk factors, including insect species unique to Australia that have the potential to be LSDV vectors, as well as take into account the effects of climate change on environmental suitability for LSDV vectors. These multi-criteria models will assist in the development of risk-based surveillance of LSD in Australia if an incursion were to occur. As the complexity of these models increases, the development of country-specific biosecurity digital twins may be plausible. A biosecurity digital twin would be derived from compiled epidemiological models and surveillance data and would enable the virtual assessment of disease risks and/or the effectiveness of interventions to better prepare for exotic disease incursions.

## Data Availability

All data supporting the findings of this review are included in the main article and the supplementary materials.

## Supplementary Materials

Supplementary 1 Contains details of search strategy and data extraction process.

Supplementary 2 Contains a list of literature included in this review.
