# Supplementary material for: Epidemiological Risk Factors and Modelling Approaches for Risk Assessment of Lumpy Skin Disease Virus Introduction and Spread: Methodological Review and Implications for Risk-Based Surveillance in Australia
Source: Transbound Emerg Dis. 2024 May 2;2024:3090226. doi: 10.1155/2024/3090226 (PMC12016705; doi:10.1155/2024/3090226)
Supplement: Supplementary 1 — Contains details of search strategy and data extraction process. [file 3090226.f1.docx]

Epidemiological risk factors and modelling approaches for risk assessment of lumpy skin disease virus introduction and spread: methodological review and implications for risk-based surveillance in Australia

# Supplementary file 1: Details of search strategy and data extraction process

## PICO process

For the topic of this literature review, the component of comparison was not applicable because this review sought to provide a comprehensive overview of all relevant literature.

- **Population or problem:** Cattle, LSDV, lumpy skin disease, bovine, cow
- **Intervention or exposure:** control, prevention, transmission, risk factors, weather, environment, socio-economic status, biosecurity, mosquito, vector-borne
- **Outcome:** infection, transmission, mortality, morbidity, distribution

## Search strategy

### PubMed

((LSDV[Title/Abstract]) OR (lumpy skin disease[Title/Abstract])) AND ((cattle[Title/Abstract]) OR (bovine[Title/Abstract]) OR (cow[Title/Abstract])) AND ((control) OR (prevention) OR (transmission) OR (risk factors) OR (weather) OR (environment) OR (socio-economic status) OR (biosecurity) OR (mosquito) OR (vector-borne)) AND ((infection) OR (transmission) OR (mortality) OR (morbidity) OR (distribution)) NOT (clinical trial[Title/Abstract]) NOT (randomized control trial[Title/Abstract]) NOT (vaccine[Title/Abstract]) NOT (vaccination[Title/Abstract]) NOT (genomic [Title/Abstract]) NOT (genome [Title/Abstract]) NOT (phylogeny [Title/Abstract]) NOT (biochemical [Title/Abstract])

### Scopus

TITLE-ABS-KEY((LSDV) OR ("lumpy skin disease")) AND TITLE-ABS-KEY((cattle) OR (bovine) OR (cow)) AND ALL(((control) OR (prevention) OR (transmission) OR (risk factors) OR (weather) OR (environment) OR ("socio-economic status") OR (biosecurity) OR (mosquito) OR ("vector-borne")) AND ((infection) OR (transmission) OR (mortality) OR (morbidity) OR (distribution))) AND NOT TITLE-ABS-KEY("clinical trial") AND NOT TITLE-ABS-KEY("randomized control trial") AND NOT TITLE-ABS-KEY(vaccine) AND NOT TITLE-ABS-KEY(vaccination) AND NOT TITLE-ABS-KEY(genomic) AND NOT TITLE-ABS-KEY(genome) AND NOT TITLE-ABS-KEY(Phylogeny) AND NOT TITLE-ABS-KEY(biochemical)

### Web of Science

(TI=(LSDV) OR TI=("lumpy skin disease") OR AB=(LSDV) OR AB=("lumpy skin disease") OR AK=(LSDV) OR AK=("lumpy skin disease")) AND (TI=(cattle) OR TI=(bovine) OR TI=(cow) OR AB=(cattle) OR AB=(bovine) OR AB=(cow) OR AK=(cattle) OR AK=(bovine) OR AK=(cow)) AND (ALL=(control) OR ALL=(prevention) OR ALL=(transmission) OR ALL=(risk factors) OR ALL=(weather) OR ALL=(environment) OR ALL=("socio-economic status") OR ALL=(biosecurity) OR ALL=(mosquito) OR ALL=("vector-borne")) AND (ALL=(infection) OR ALL=(transmission) OR ALL=(mortality) OR ALL=(morbidity) OR ALL=(distribution)) NOT ALL=("clinical trial") NOT ALL=("randomized control trial") NOT ALL=(vaccine) NOT ALL=(vaccination) NOT ALL=(genomic) NOT ALL=(genome) NOT ALL=(Phylogeny) NOT ALL=(biochemical)

### Europe PMC

(TITLE:"LSDV" OR TITLE:"lumpy skin disease" OR ABSTRACT:"LSDV" OR ABSTRACT:"lumpy skin disease" OR KW:"LSDV" OR KW:"lumpy skin disease") AND (TITLE:"cattle" OR TITLE:"bovine" OR TITLE:"cow" OR ABSTRACT:"cattle" OR ABSTRACT:"bovine" OR ABSTRACT:"cow" OR KW:"cattle" OR KW:"bovine" OR KW:"cow") AND ("control" OR "prevention" OR "transmission" OR "risk factors" OR "weather" OR "environment" OR "socio-economic status" OR "biosecurity" OR "mosquito" OR "vector-borne") AND ("infection" OR "transmission" OR "mortality" OR "morbidity" OR "distribution") NOT "clinical trial" NOT "randomized control trial" NOT "vaccine" NOT "vaccination" NOT "genomic" NOT "genome" NOT "Phylogeny" NOT "biochemical"

## Data extraction and storage of results

Key information from selected literature was summarised using a predefined MS Excel spreadsheet to allow for easy comparison of extracted information. The information extracted from all articles which underwent full review were under five main themes: global and regional epidemiology of LSD, risk factors for LSDV exposure and infection in cattle in endemic countries, risk factors influencing probability of introduction (illegal processes)/importation (legal processes) and probability of detection of infected cattle from endemic countries to LSD-free countries, surveillance strategies that are being implemented in LSD-free countries, and epidemiological approaches for LSDV decision-support.
