# Supplementary material for: Epidemiological Risk Factors and Modelling Approaches for Risk Assessment of Lumpy Skin Disease Virus Introduction and Spread: Methodological Review and Implications for Risk-Based Surveillance in Australia
Source: Transbound Emerg Dis. 2024 May 2;2024:3090226. doi: 10.1155/2024/3090226 (PMC12016705; doi:10.1155/2024/3090226)
Supplement: Supplementary 2 — Contains a list of literature included in this review. [file 3090226.f2.docx]

| **Reference number in manuscript** | **Reference citation** | **Topics covered** |
| --- | --- | --- |
| **1** | Tuppurainen ES, Oura CA. Review: lumpy skin disease: an emerging threat to Europe, the Middle East and Asia. Transbound Emerg Dis. 2012;59(1):40-8. | Review of LSD outbreak situation in Middle East, Europe, and Asia, and recent developments in testing and control measures. |
| **4** | Lu G, Xie J, Luo J, Shao R, Jia K, Li S. Lumpy skin disease outbreaks in China, since 3 August 2019. Transbound Emerg Dis. 2021;68(2):216-9. | Case reports of first LSD outbreaks in China in 2019-2020. |
| **5** | Mat B, Arikan MS, Akin AC, Çevrimli MB, Yonar H, Tekindal MA. Determination of production losses related to lumpy skin disease among cattle in Turkey and analysis using SEIR epidemic model. BMC Vet Res. 2021;17(1):300. | SEIR model predicting financial losses associated with LSD outbreaks in Turkey. |
| **6** | Shah SH, Khan M. Lumpy skin disease emergence in Pakistan, a new challenge to the livestock industry. J Vet Sci. 2022;23(5):e77. | Letter to editor on first LSD outbreak in Pakistan in 2021. |
| **7** | Tuppurainen E, Oura C. Lumpy skin disease: An African cattle disease getting closer to the EU. Veterinary Record. 2014;175(12):300-1. | Editorial on spread of LSD approaching EU and recommendations for prevention activities. |
| **8** | Welay GM, Tedla DG, Teklu GG, Weldearegay SK, Shibeshi MB, Kidane HH, et al. A preliminary survey of major diseases of ruminants and management practices in Western Tigray province, northern Ethiopia. BMC Vet Res. 2018;14(1):293. | Survey of farmers livestock management practices in Ethiopia in relation to major diseases including LSD. |
| **10** | Chihota CM, Rennie LF, Kitching RP, Mellor PS. Mechanical transmission of lumpy skin disease virus by Aedes aegypti (Diptera: Culicidae). Epidemiol Infect. 2001;126(2):317-21. | Experimental testing of mechanical transmission of LSDV from Ae aegypti mosquitoes to susceptible cattle. |
| **11** | Sanz-Bernardo B, Haga IR, Wijesiriwardana N, Basu S, Larner W, Diaz AV, et al. Quantifying and Modeling the Acquisition and Retention of Lumpy Skin Disease Virus by Hematophagus Insects Reveals Clinically but Not Subclinically Affected Cattle Are Promoters of Viral Transmission and Key Targets for Control of Disease Outbreaks. Journal of virology. 2021;95(9):e02239-20. | Experimental study in UK on mechanical transmission of LSDV of four biting insect vectors to and from clinical and subclinical cattle. |
| **13** | Beard PM. Lumpy skin disease: A direct threat to Europe. Veterinary Record. 2016;178(22):557-8. | Summary of geographical spread of LSD from Africa to Middle East and East Europe and risk to Europe. |
| **14** | Ratyotha K, Prakobwong S, Piratae S. Lumpy skin disease: A newly emerging disease in Southeast Asia. Vet World. 2022;15(12):2764-71. | Review of epidemiology and clinical features of LSDV and summary of LSD situation in Southeast Asia. |
| **27** | Abera Z, Degefu H, Gari G, Kidane M. Sero-prevalence of lumpy skin disease in selected districts of West Wollega zone, Ethiopia. BMC Vet Res. 2015;11:135. | Seroprevalence of LSD in cattle in Ethiopia. Associations with LSD seroprevalence and Age, Sex, Breed, and Altitude, as well as districts of Ethiopia. |
| **28** | Abutarbush SM, Ababneh MM, Al Zoubi IG, Al Sheyab OM, Al Zoubi MG, Alekish MO, et al. Lumpy Skin Disease in Jordan: Disease Emergence, Clinical Signs, Complications and Preliminary-associated Economic Losses. Transbound Emerg Dis. 2015;62(5):549-54. | Case reports of LSD in dairy cattle in Jordan. Epidemiological profile of LSD associated morbidity, mortality, case fatality, as well as clinical signs including decreased weight, decrease milk production. |
| **29** | Albayrak H, Ozan E, Kadi H, Cavunt A, Tamer C, Tutuncu M. Molecular detection and seasonal distribution of lumpy skin disease virus in cattle breeds in Turkey. Med Weter. 2018;74(3):175-8. | Epidemiology of LSD in cattle in Turkey. Chi square results for seasonality of cases in different provinces, LSD prevalence compared to age, sex, breed. |
| **30** | Alemayehu G, Zewde G, Admassu B. Risk assessments of lumpy skin diseases in Borena bull market chain and its implication for livelihoods and international trade. Tropical Animal Health and Production. 2013;45(5):1153-9. | Qualitative risk assessment of LSD introduction through Borena bulls in Ethiopia using OIE assessment model. Two exposure pathways: blood-feeding arthropods and infected equipment. |
| **31** | Anwar A, Na-Lampang K, Preyavichyapugdee N, Punyapornwithaya V. Lumpy Skin Disease Outbreaks in Africa, Europe, and Asia (2005–2022): Multiple Change Point Analysis and Time Series Forecast. Viruses. 2022;14(10). | ARIMA and NNAR time series forecast models of LSD outbreaks in Africa, Europe and Asia using WOAH LSD report data from 2005-2022 to predict 2022-2024. |
| **32** | Ardestani EG, Mokhtari A. Modeling the lumpy skin disease risk probability in central Zagros Mountains of Iran. Preventive Veterinary Medicine. 2020;176. | Maximum entropy ecological niche modelling (MaxEnt) for probability of LSD outbreak in Iran based on environmental variables. |
| **33** | Arjkumpa O, Suwannaboon M, Boonrawd M, Punyawan I, Laobannu P, Yantaphan S, et al. First emergence of lumpy skin disease in cattle in Thailand, 2021. Transbound Emerg Dis. 2021;68(6):3002-4. | First cases of LSD in Thailand in 2021. Philogenetic analysis of LSDV isolates. |
| **34** | Arjkumpa O, Suwannaboon M, Boonrod M, Punyawan I, Liangchaisiri S, Laobannue P, et al. The First Lumpy Skin Disease Outbreak in Thailand (2021): Epidemiological Features and Spatio-Temporal Analysis. Front Vet Sci. 2021;8:799065. | Spatial, temporal, and spatio-temporal cluster analysis of LSD outbreaks in Thailand. |
| **35** | Azeem S, Sharma B, Shabir S, Akbar H, Venter E. Lumpy skin disease is expanding its geographic range: A challenge for Asian livestock management and food security. Vet J. 2022;279:105785. | Summary of LSD outbreaks in Asian countries and geographical spread. Possibility of migratory birds carrying LSDV infected ticks. |
| **36** | Babiuk S, Bowden TR, Boyle DB, Wallace DB, Kitching RP. Capripoxviruses: an emerging worldwide threat to sheep, goats and cattle. Transbound Emerg Dis. 2008;55(7):263-72. | Review of capripoxviruses including LSDV. |
| **37** | Davies FG. Lumpy skin disease, an African capripox virus disease of cattle. Br Vet J. 1991;147(6):489-503. | Review of LSDV aetiology, clinical signs, pathology, diagnosis, epizootiology, prevention, control and eradication. |
| **38** | Dubie T, Hussen Abegaz F, Dereje B, Negash W, Hamid M. Seroprevalence and Associated Risk Factors of Lumpy Skin Disease of Cattle in Selected Districts of Afar Region, Ethiopia. Vet Med (Auckl). 2022;13:191-9. | Cross-sectional study of LSD seroprevalence in Ethiopia and risk factors including, age, sex, herd size, and district. |
| **39** | Farra D, De Nardi M, Lets V, Holopura S, Klymenok O, Stephan R, et al. Qualitative assessment of the probability of introduction and onward transmission of lumpy skin disease in Ukraine. Microbial Risk Analysis. 2022;20. | Qualitative risk assessment of LSD introduction into Ukraine and onward transmission using various transmission pathways based on available evidence and expert workshop conclusions. |
| **40** | Gale P, Kelly L, Snary EL. Qualitative assessment of the entry of capripoxviruses into Great Britain from the European Union through importation of ruminant hides, skins and wool. Microbial Risk Analysis. 2016;1:13-8. | Qualitative risk assessment of importing LSD infected hides and skins into UK based on probabilities of infection in source herds in Europe. |
| **41** | Gari G, Waret-Szkuta A, Grosbois V, Jacquiet P, Roger F. Risk factors associated with observed clinical lumpy skin disease in Ethiopia. Epidemiol Infect. 2010;138(11):1657-66. | Cross-sectional study exploring associations between LSD in cattle in Ethiopia and risk factors including terrain, communal/private grazing/watering, and new cattle introduction. |
| **42** | Gubbins S. Using the basic reproduction number to assess the risk of transmission of lumpy skin disease virus by biting insects. Transbound Emerg Dis. 2019;66(5):1873-83. | Modelling of reproduction ratio for five species of biting insects using previous studies findings for parameterisation. |
| **43** | Hailu B, Tolosa T, Gari G, Teklue T, Beyene B. Estimated prevalence and risk factors associated with clinical Lumpy skin disease in north-eastern Ethiopia. Prev Vet Med. 2014;115(1-2):64-8. | Cross-sectional study. Estimates of LSD prevalence in Ethiopia. Multivariate model of risk factors of LSD: herd size, communal grazing/watering, introduction of new cattle. |
| **44** | Hässig M, Meier AB, Braun U, Urech Hässig B, Schmidt R, Lewis F. Cattle movement as a risk factor for epidemics. Schweiz Arch Tierheilkd. 2015;157(8):441-8. | Description of Swiss cattle movement database. Measurement of cattle movement during incubation period of LSD (28 days) between January 2011 and January 2012. |
| **45** | Imran M, Hashmi AH, Khalique F, Iqbal MZ. Lumpy Skin Disease Emerging Problem in Pakistan. Journal of Clinical Cases & Reports. 2023;6(S11):128-132. | Review of emergence of LSD in Pakistan in late 2021 / early 2022. |
| **46** | Ince ÖB, Çakir S, Dereli MA. Risk analysis of lumpy skin disease in Turkey. Indian Journal of Animal Research. 2016;50(6):1013-7. | Risk analysis of LSD in Turkey exploring release and exposure pathways. |
| **47** | Issimov A, Kushaliyev K, Abekeshev N, Molla W, Rametov N, Bayantassova S, et al. Risk factors associated with lumpy skin disease in cattle in West Kazakhstan. Prev Vet Med. 2022;207:105660. | Cross-sectional study of LSD in West Kazakhstan. Multivariable model of risk factors: Herd size, purchasing animals, selling animals during LSD outbreak. |
| **48** | Issimov A, Kutumbetov L, Orynbayev MB, Khairullin B, Myrzakhmetova B, Sultankulova K, et al. Mechanical transmission of lumpy skin disease virus by stomoxys spp (Stomoxys calsitrans, stomoxys sitiens, stomoxys indica), diptera: Muscidae. Animals. 2020;10(3). | Experimental study of mechanical transmission of LSDV by three Stomoxys species. First to demonstrate mechanical transmission of LSDV by three Stomoxys species in Kazakhstan. |
| **49** | Kahana-Sutin E, Klement E, Lensky I, Gottlieb Y. High relative abundance of the stable fly Stomoxys calcitrans is associated with lumpy skin disease outbreaks in Israeli dairy farms. Med Vet Entomol. 2017;31(2):150-60. | Model of measured biting insect populations and rainfall compared to temporal distribution of LSDV outbreaks in dairy farms in Israel. |
| **50** | Khatri G, Rai A, Aashish, Shahzaib, Hyder S, Priya, et al. Epidemic of lumpy skin disease in Pakistan. Vet Med Sci. 2023. | Summary of LSD situation in Pakistan with recommendations for control measures. |
| **51** | Klausner Z, Fattal E, Klement E. Using Synoptic Systems' Typical Wind Trajectories for the Analysis of Potential Atmospheric Long-Distance Dispersal of Lumpy Skin Disease Virus. Transbound Emerg Dis. 2017;64(2):398-410. | Modelling of wind trajectories for possible transportation of LSDV vectors into Israel during two previous outbreaks. |
| **52** | Koleci X, Lilo A, Papa S, Margariti K, van Roon A, Santman-Berends I, et al. An Overview of Current Approaches and Challenges to the Control of Endemic Infectious Cattle Diseases in Albania. Front Vet Sci. 2021;8:671873. | Review of LSD control programmes in Albania. |
| **53** | Limon G, Gamawa AA, Ahmed AI, Lyons NA, Beard PM. Epidemiological Characteristics and Economic Impact of Lumpy Skin Disease, Sheeppox and Goatpox Among Subsistence Farmers in Northeast Nigeria. Front Vet Sci. 2020;7:8. | Modelling of economic impact of LSD outbreak in Nigeria. |
| **54** | Lubinga JC, Clift SJ, Tuppurainen ES, Stoltsz WH, Babiuk S, Coetzer JA, et al. Demonstration of lumpy skin disease virus infection in Amblyomma hebraeum and Rhipicephalus appendiculatus ticks using immunohistochemistry. Ticks Tick Borne Dis. 2014;5(2):113-20. | Experimental study of infection and transmission of LSDV in two species of ticks: R. appendiculatus (mechanical, intrastadial, transstadial), and A. hebraeum (mechanical, intrastadial, transstadial) in South Africa. |
| **55** | Lubinga JC, Tuppurainen ES, Coetzer JA, Stoltsz WH, Venter EH. Evidence of lumpy skin disease virus over-wintering by transstadial persistence in Amblyomma hebraeum and transovarial persistence in Rhipicephalus decoloratus ticks. Exp Appl Acarol. 2014;62(1):77-90. | Experimental study demonstrating over-wintering of LSDV in R. appendiculatus and A. hebraeum in South Africa. |
| **56** | Lubinga JC, Tuppurainen ES, Mahlare R, Coetzer JA, Stoltsz WH, Venter EH. Evidence of transstadial and mechanical transmission of lumpy skin disease virus by Amblyomma hebraeum ticks. Transbound Emerg Dis. 2015;62(2):174-82. | Experimental study demonstrating transtadial transmission of LSDV in A. hebraeum ticks for first time. |
| **57** | Lv DH, Zhai SL, Wei WK, Zhai Q, Wen XH, Chen QL. Threat of lumpy skin disease to the Chinese cattle industry. Vet Rec. 2021;188(8):315-6. | Discussion of LSD vaccination and interventions during two outbreaks in China between 2019 and 2020 and their effectiveness. |
| **58** | Machado G, Korennoy F, Alvarez J, Picasso-Risso C, Perez A, VanderWaal K. Mapping changes in the spatiotemporal distribution of lumpy skin disease virus. Transbound Emerg Dis. 2019;66(5):2045-57. | Ecological niche modelling. Spatio-temporal analysis of LSDV risk in eastern Europe using environmental factors including temperature, precipitation, and wind speed. |
| **59** | Magori-Cohen R, Louzoun Y, Herziger Y, Oron E, Arazi A, Tuppurainen E, et al. Mathematical modelling and evaluation of the different routes of transmission of lumpy skin disease virus. Vet Res. 2012;43(1):1. | Transmission model of LSDV infection among cattle in farm in Israel based on direct and indirect contact with R0 for different transmission routes, including indirect transmission (flying insects), direct transmission (sharing water source), transmission during milking. |
| **60** | Maw MT, Khin MM, Hadrill D, Meki IK, Settypalli TBK, Kyin MM, et al. First Report of Lumpy Skin Disease in Myanmar and Molecular Analysis of the Field Virus Isolates. Microorganisms. 2022;10(5). | Case report of first LSD outbreaks in Myanmar and phylogenetic analysis of LSDV isolates. |
| **61** | Mercier A, Arsevska E, Bournez L, Bronner A, Calavas D, Cauchard J, et al. Spread rate of lumpy skin disease in the Balkans, 2015-2016. Transbound Emerg Dis. 2018;65(1):240-3. | Spatial and temporal analysis of spread of LSD in Balkans with estimate of LSD spread rate. |
| **62** | Molla W, de Jong MCM, Frankena K. Temporal and spatial distribution of lumpy skin disease outbreaks in Ethiopia in the period 2000 to 2015. BMC Vet Res. 2017;13(1):310. | Spatial temporal patterns of LSD outbreaks in Ethiopia between 2000-2015 and ARIMA time series forecast for 2016-2018. |
| **63** | Molla W, Frankena K, MCM DEJ. Transmission dynamics of lumpy skin disease in Ethiopia. Epidemiol Infect. 2017;145(13):2856-63. | SIR transmission model of LSD in Ethiopia with reproduction ratio of LSD comparison between crop-livestock production systems and intensive production systems. |
| **64** | Morgan KL. Allergic wheals, abortion and lumpy skin disease. Veterinary Record. 2016;178(20):508-9. | Summary of LSD epidemiological situation in Europe and recommendations for early detection. |
| **65** | Namazi F, Khodakaram Tafti A. Lumpy skin disease, an emerging transboundary viral disease: A review. Vet Med Sci. 2021;7(3):888-96. | Overview of global epidemiology of LSD including pathology, transmission, risk factors, economic impacts, diagnosis, prevention, and control. |
| **66** | Ocaido M, Otim CP, Kakaire D. Impact of major diseases and vectors in smallholder cattle production systems in different agro-ecological zones and farming systems in Uganda. Livestock Research for Rural Development. 2009;21(9). | Prevalence of diseases including LSD among cattle in smallholder cattle farms in Uganda and estimates of associated economic losses. |
| **67** | Ochwo S, VanderWaal K, Munsey A, Ndekezi C, Mwebe R, Okurut ARA, et al. Spatial and temporal distribution of lumpy skin disease outbreaks in Uganda (2002-2016). BMC Vet Res. 2018;14(1):174. | Identification of spatial and spatio-temporal LSD hotspots in Uganda. |
| **68** | Ochwo S, VanderWaal K, Munsey A, Nkamwesiga J, Ndekezi C, Auma E, et al. Seroprevalence and risk factors for lumpy skin disease virus seropositivity in cattle in Uganda. BMC Vet Res. 2019;15(1):236. | Associations between LSD seroprevalence in Uganda and risk factors including precipitation, management practices, and communal water sources. |
| **69** | Pandey N, Hopker A, Prajapati G, Rahangdale N, Gore K, Sargison N. Observations on presumptive lumpy skin disease in native cattle and Asian water buffaloes around the tiger reserves of the central Indian highlands. N Z Vet J. 2022;70(2):101-8. | Clinical features of LSD in bovines in 32 villages in India. |
| **70** | Paslaru AI, Maurer LM, Vögtlin A, Hoffmann B, Torgerson PR, Mathis A, et al. Putative roles of mosquitoes (Culicidae) and biting midges (Culicoides spp.) as mechanical or biological vectors of lumpy skin disease virus. Medical and Veterinary Entomology. 2022;36(3):381-9. | Experimental study of LSDV retention in mosquitoes and biting midges. |
| **71** | Paslaru AI, Verhulst NO, Maurer LM, Brendle A, Pauli N, Vögtlin A, et al. Potential mechanical transmission of Lumpy skin disease virus (LSDV) by the stable fly (Stomoxys calcitrans) through regurgitation and defecation. Curr Res Insect Sci. 2021;1:100007. | Experimental study of LSDV retention in Stomoxys calcitrans stable flies. Presence of S. calcitrans in Swiss farms. |
| **72** | Punyapornwithaya V, Seesupa S, Phuykhamsingha S, Arjkumpa O, Sansamur C, Jarassaeng C. Spatio-temporal patterns of lumpy skin disease outbreaks in dairy farms in northeastern Thailand. Front Vet Sci. 2022;9:957306. | Spatio-temporal cluster analysis of LSD outbreaks in Thailand in 2021. |
| **73** | Rouby SR, Hussein KH, Aboelhadid SM, El-Sherif AM. Role of rhipicephalus annulatus tick in transmission of lumpy skin disease virus in naturally infected cattle in Egypt. Advances in Animal and Veterinary Sciences. 2017;5(4):185-91. | Experimental testing of transovarial transmission of LSDV in R. annulatus ticks taken from naturally infected cattle in Egypt. |
| **74** | Sabirovic M, Raw L, Rogers A, Lock F, Hall S, Elliott H, et al. International disease monitoring, July to September 2006. Veterinary Record. 2006;159(21):695-8. | Case report confirming localised LSD outbreak in Israel in 2006. |
| **75** | Saegerman C, Bertagnoli S, Meyer G, Ganière JP, Caufour P, De Clercq K, et al. Risk of introduction of lumpy skin disease in France by the import of vectors in animal trucks. PLoS One. 2018;13(6):e0198506. | QIRA model risk analysis of introduction of LSD infected stable flies through animal trucks entering France and travelling to farms and slaughterhouses. |
| **76** | Saegerman C, Bertagnoli S, Meyer G, Ganière JP, Caufour P, De Clercq K, et al. Risk of introduction of Lumpy Skin Disease into France through imports of cattle. Transbound Emerg Dis. 2019;66(2):957-67. | QIRA model risk analysis of importation of LSD infected cattle into France sent to farms or slaughterhouses in summer and winter months. |
| **77** | Selim A, Manaa E, Khater H. Seroprevalence and risk factors for lumpy skin disease in cattle in Northern Egypt. Trop Anim Health Prod. 2021;53(3):350. | Key risk factors for seroprevalence of LSD in cattle in Egypt, including cattle age and use of communal water source. |
| **78** | Şevik M, Doğan M. Epidemiological and Molecular Studies on Lumpy Skin Disease Outbreaks in Turkey during 2014-2015. Transbound Emerg Dis. 2017;64(4):1268-79. | GLM exploring association between LSD in cattle in Turkey and risk factors including herd size and farm's being near lakes. Phylogenetic analysis of LSD isolates collected from field. |
| **79** | Sohier C, Haegeman A, Mostin L, De Leeuw I, Campe WV, De Vleeschauwer A, et al. Experimental evidence of mechanical lumpy skin disease virus transmission by Stomoxys calcitrans biting flies and Haematopota spp. horseflies. Sci Rep. 2019;9(1):20076. | Experimental study on mechanical transmission of LSD by stable flies and horseflies to cattle. |
| **80** | Tageldin MH, Wallace DB, Gerdes GH, Putterill JF, Greyling RR, Phosiwa MN, et al. Lumpy skin disease of cattle: an emerging problem in the Sultanate of Oman. Trop Anim Health Prod. 2014;46(1):241-6. | First confirmation of presence and seroprevalence of LSDV in cattle in Sultanate of Oman. |
| **81** | Tuppurainen ES, Lubinga JC, Stoltsz WH, Troskie M, Carpenter ST, Coetzer JA, et al. Evidence of vertical transmission of lumpy skin disease virus in Rhipicephalus decoloratus ticks. Ticks Tick Borne Dis. 2013;4(4):329-33. | Experimental study first reporting transovarial transmission of LSD in R. decoloratus ticks. |
| **82** | Tuppurainen ES, Stoltsz WH, Troskie M, Wallace DB, Oura CA, Mellor PS, et al. A potential role for ixodid (hard) tick vectors in the transmission of lumpy skin disease virus in cattle. Transbound Emerg Dis. 2011;58(2):93-104. | Experimental study confirming transstadial and transovarial transmission of LSDV by R. decoloratus ticks and mechanical transmission by R. appendiculatus and A. hebraeum ticks. |
| **83** | Tuppurainen ES, Venter EH, Coetzer JA, Bell-Sakyi L. Lumpy skin disease: attempted propagation in tick cell lines and presence of viral DNA in field ticks collected from naturally-infected cattle. Ticks Tick Borne Dis. 2015;6(2):134-40. | Study confirming presence of LSDV DNA in hard ticks collected in field from naturally infected cattle in South Africa and Egypt. |
| **84** | Yeruham I, Nir O, Braverman Y, Davidson M, Grinstein H, Haymovitch M, et al. Spread of lumpy skin disease in Israeli dairy herds. Vet Rec. 1995;137(4):91-3. | Discussion of first LSD outbreaks in Israel and possible modes of introduction including stable flies carried by wind. |
